# Hospital and Institutional News

**Published:** 1917-10-27

**Authors:** 


### HOSPITAL AND INSTITUTIONAL NEWS.

### THE MEDICAL WAR MUSEUM.

Soon after the commencement of the war it was felt that a collection should be made of specimens illustrative of the injuries received in battle, and the Army Medical Department arranged with the Royal College of Surgeons of England that the Museum staff and the workrooms of the College should be placed at the disposal of the Army Council. Large consignments of pathological specimens have been received from time to time at the College from abroad, and also from the military hospitals of this country, and a large number of the specimens have been dissected and mounted by the Pathological Curator, Mr. S. G. Shattock, and now an exhibition of these specimens has been arranged at the College of Surgeons. As stated in The Hospital (October 20, page 43), this exhibition was opened on October 11 by Sir Alfred Keogh, the Director-General of the Army Medical Service. Three rooms of the Hunterian Museum have been arranged for the exhibition of the specimens, and hundreds of examples of injury of the various organs and structures of the body are shown, and for comparing and contrasting with these there are specimens derived from former wars showing the lesions produced by the less severe projectiles then in use. In addition to the pathological specimens are to be seen examples of helmets and breast-plates and samples of the paper bandages and lint taken from captured German dressing stations. Very striking specimens are those showing the injuries to the lungs caused by "gassing." The most remarkable specimen of all is the heart of a flying officer, who, although his heart had been severely lacerated, was able to bring his aeroplane to earth. This is merely an interim exhibition, but it is hoped that it will prove useful to surgeons engaged in treating the wounded by assisting them in dealing with the cases under their charge. When the war ends, and the exhibition is complete, then the store of knowledge should prove of the greatest value to those who come after us, unless, as some promise us, this war will be the last.

### MINISTRY OF HEALTH BILL SHELVED.

A deputation, representing twelve million insured persons, was received on October 11 by the Prime Minister, who was accompanied by Lord Rhondda, Mr. Hayes Fisher, Sir Edwin Cornwall, and others. The object of the deputation was to urge (1) the necessity of the early establishment of a Ministry of Health; (2) the early passage into law of a Bill for the simplification of National Health Insurance administration; and (3) the necessity of further Exchequer grants for the purposes of National Insurance. The deputation was introduced by the Right Hon. J. H. Thomas, M.P., who very emphatically made it clear that a Ministry of Health which is in any way identified with the Local Government Board would not be acceptable to insured persons under the National Insurance Acts nor to the trade unions. The Prime Minister, in his reply, referred sympathetically to the attractions of the proposal to set up a Ministry of Health, and agreed that stricter attention to the health of the community would be necessary in order to repair the ravages of war. But while the general principle might be accepted, the details of any measure designed to give effect thereto were bound to produce a clash of interests. In these circumstances there was no alternative but to defer the reform of the health administration until after the war. The Ministry of Health Bill, prepared by National Insurance organisations, is therefore shelved for the time being, as also, apparently, is the scheme which Lord Rhondda had proposed. This does not necessarily mean that the Ministry will not ultimately materialise, for, as the Prime Minister indicated, it is by work and not merely by talk that reform is to be accomplished. Lord Rhondda is undoubtedly in earnest in the matter, and so also are the leaders of the National Insurance organisations. If the Ministry of Health is not actually in being before the end of the war there is no reason why good, honest work should not prepare the way for its establishment immediately after peace is declared.

### SIMPLIFICATION OF NATIONAL INSURANCE ADMINISTRATION.

With regard to the simplification of the administration of National Insurance the necessary investigation has already been made, and early legislation on the basis of the recommendations of the Ryan Committee is promised. The Prime Minister made it clear, however, that the resources of the nation must be husbanded, and that no obligations must be incurred beyond the strictest and sternest necessities of the hour, and consequently very little could be expected from the Treasury in the way of extra grants for the purposes of National Insurance. When the war is over the whole question will have to be reconsidered, and the Prime Minister hoped that when this is done it will be reconsidered in a wide and broad spirit.

### THE ELIMINATION OF TYPHOID.

In the British Armies the number of cases of typhoid fever has enormously decreased, mainly owing to the prophylactic use of antityphoid vaccine; and this is also true of the allied diseases "A and B Paratyphoid," for in the preparation of the vaccine the germs of all these diseases are employed. A similar happy result has followed the use of antityphoid vaccine in the French Army. In that Army preventive vaccination against typhoid was commenced in February 1915, and since then the percentage of typhoid-fever cases has steadily diminished, until a year later it was less than 1 per 1,000. According to the latest returns, the frequency of typhoid has become only .063 per 1,000. That figure refers to the frequency of the cases; but the mortality figures show even more clearly the value of the vaccine, for even when an attack does occur after injection, the body is so protected that the attack is very mild, and death is extremely rare. The death-rate is only 3 per 1,000,000. It has been calculated that the use of the vaccine has saved more than two hundred thousand lives in the French Army. In fact, so great has been its value that among the troops the frequency of typhoid has been only one-seventh of that met with in peace-time, and the mortality has been even less still.

### THE CANADIAN HOSPITALS IN FRANCE.

The construction of the new hospital for the Canadian Red Cross Society at Joinville in France is progressing satisfactorily, and at the end of the year it is hoped to transfer to it the medical staff from Troyes. A few days ago Colonel G. Beauchamp and Lieut.-Colonel Rhéaume, of No. 6 Canadian General Hospital, met Lieut.-Colonel Blaylock, Assistant Commissioner of the Canadian Red Cross Society, and Mr. Skipper, the architect, in Paris, and together they proceeded to inspect the work already done. Colonel Beauchamp and Lieut.-Colonel Rhéaume, while in Paris, paid a visit to the school which has been established for the re-education of wounded French soldiers at the Agricultural School of Grignon. Lieut.-Colonel Le Bel, Director of the Canadian Hospital at St. Cloud, who has rendered noteworthy service in the creation of travelling surgical units at the Front, has been honoured by the French Government, which has created him a Chevalier of the Legion of Honour. Captain Marcille, of the St. Cloud Hospital, has recently gone to the French Front in charge of a field ambulance of six doctors, twenty nurses, and fifty men.

### THE NATIONAL MEDICAL UNION.

At a meeting of the Council of the National Medical Union, held at 346 Strand, London, on Friday, October 19, and presided over by Mr. V. T. Greenyer, F.R.C.S., Chairman of Council, the following resolutions were agreed to unanimously:—

(1) That the National Medical Union advises its members and all other non-panel medical practitioners to refrain from attending discharged disabled soldiers and sailors under "the special arrangements" of the Insurance Commissioners, but encourages treatment in their private professional capacity. (2) That the National Medical Union regrets that it has been considered necessary to replace British medical men capable and desirous of retaining their appointments in the war hospitals by foreigners holding foreign qualifications. (3) That the Council of the National Medical Union deprecates that section of the General Order of the Local Government Board which instructs and allows the diagnosis and notification of measles and German measles by the lay public.

### NEW PROPOSALS FOR DISABLED SOLDIERS.

Mr. Hodge, the Minister of Pensions, is nothing if not progressive, and his new suggestions and regulations for the amelioration of the lot of the disabled soldier will meet with the heartiest approval. The compulsory inclusion of disabled men upon every local committee is an act of common justice, and the reluctance of the committees themselves to comply with the suggestion of the Pensions Ministry that they should be so included is difficult to understand. Further, Mr. Hodge has found in the course of an extended inspection of military hospitals that many of the artificial limbs now being supplied are not as good as they ought to be, and are, indeed, in some cases an actual danger to the user. To remedy this a sum of £10,000 has been voted by the Treasury to found an experimental factory, where the highest mechanical and surgical skill will be enlisted to design the most perfect artificial limbs that human ingenuity can devise. The finding of occupations for permanently disabled men is one which continues to engage Mr. Hodge's benevolent activities, and the manifold openings which present themselves to discharged men after they have undergone the preparatory period of training is a striking proof of what can be achieved by a little foresight and organisation. In a case quoted by Mr. Hodge, and one can only hope that it is one of many, a man who before the war was earning 18s. a week as an agricultural labourer, is now in receipt of £2 5s., plus his pension, at an aeroplane factory. The men who have risked their all in fighting for us must have no cause to complain of our want of generosity.

### SOLDIERS AND ASYLUMS.

It is welcome hearing that yet another grievance in the treatment of men discharged from the Army on account of insanity has, somewhat tardily, been removed. Formerly, as now, these men were divided into two classes, Service and non-Service patients, according as to whether their disability could be directly attributed to the conditions of service or ante-dated enlistment. All men discharged for insanity and placed in asylums become Service patients as soon as their cases have been investigated and their status determined. This takes about a fortnight, and it has now been arranged that in the interval between admission and definite allocation to the position of Service or non-Service patients all men shall be private patients paid for by the Pensions Department. They will be taken to the asylums under military escort, and be clearly distinguished from Poor-Law patients. By these provisions the real grievance of soldiers being apparently Poor-Law patients for a fortnight or so until their status could be determined has been removed. As soon as a man has been classed as a Service patient—which means all men except those who have been in asylums before the war, and whose subsequent insanity is not the result of service—he is maintained by the Department as a private paying patient and receives 2s. 6d. a week pocket-money. He wears clothes distinguishing him from Poor-Law patients and a military badge. If he has a wife and family they receive pensions on the highest scale, as if he had been killed in action. Thus the stigma of pauper insanity is entirely removed.

### THE WELFARE OF THE SOLDIER.

The existence of an International Committee for the study of all questions relating to the welfare of discharged or disabled sailors and soldiers is certainly not widely known. But such a body exists, and has just held a sitting in London. Its value, if run on the proper lines—and with Mr. Hodge at the Ministry of Pensions the British section will certainly not be the least progressive—could be incalculable. The problems connected with the disabled man's re-entry into civil life and his reinstatement as a member of the wage-earning community might well be solved by an international putting together of heads. A scheme found practicable, say, in France, would, with slight modifications, work equally well in England and America, and with facilities for organised study, new ideas would be evolved and carried out successfully in practice. The last general meeting was held in Paris last May. Next year London is the centre selected, and the delegates will be sure of hearty Government and Press co-operation.

### THE NORFOLK HOSPITAL AND THE JENNY LIND INFIRMARY.

An unfortunate position has arisen in connection with the Norfolk and Norwich Hospital and the Jenny Lind Infirmary. Soon after the beginning of the war the former institution was anxious to receive wounded soldiers, but the problem of accommodating its own patients was a difficulty. This was met when the Jenny Lind Infirmary agreed to receive the children who had formerly been among the patients at the Norfolk and Norwich Hospital. After this harmonious arrangement had been made the Jenny Lind Infirmary asked the Norfolk and Norwich Hospital for a contribution towards the work for children of which the latter institution had been relieved. The hospital agreed to give to the infirmary £250 at the half-year and another £250 in December. This was a return for the help afforded. It was not, we believe, a promise of annual obligation. Now that another year has passed the Jenny Lind has renewed its request, and the hospital board has decided that it cannot afford to grant it. Canon Pelham, in a forcible and lucid speech at the recent quarterly meeting, declared that the hospital owed a debt of honour to the infirmary, and was in duty bound to renew its former grant. Mr. Larkins said that it had to look after its own interests. A conference will have taken place, we understand, by the time these lines are in print, so it would be unbecoming to criticise till the final decision is known. We would point out, however, one significent fact which was not emphasised in the recent debate, that, namely, while the Norfolk and Norwich Hospital is in a position to receive the War Office grant in respect of the wounded in its wards, the Jenny Lind, which enabled it to receive them, is in no such fortunate position in regard to the children to whom it has opened its doors. Must not this fact be weighed in coming to a decision? Does it not make a debt of honour a positive item in the balance-sheet?

### UNDERFED STAFFS IN INFIRMARIES.

Attention has been drawn to the insufficient allowances of food for the staffs engaged at infirmaries and hospitals in the instructions of the Food Controller. It is generally felt that the amount is not enough for officers employed for long hours on arduous and responsible duties which require a great amount of skill and patience. Some Boards of Guardians have decided that their chief officers shall provide substitutes for the diminished quantities of bread, meat, and sugar; but it has been found in practice that this does not meet the difficulty, inasmuch as some of the substitutes are as difficult to obtain as the food which is limited by the Food Controller. The Southwark Board of Guardians have come to the conclusion that the officers are not obtaining sufficient food at the present time, and that this is largely owing to the fact that it is not possible to obtain any substitute for meat in the case of officers who are not vegetarians. Under these circumstances they are considering whether it would not be advisable to authorise the chiefs of their institutions to exceed the scale of two and a half pounds of meat a week at their discretion, due economy, however, to be observed. The consumption of bread and sugar is to remain the same.

### MEMORIAL BEDS AT CARDIFF.

The endowment of more memorial beds at King Edward VII.'s Hospital, Cardiff, has been announced. Mr. J. C. Gould, a generous supporter of war hospitals, has given 1,000 guineas to endow a bed in memory of Mr. Eugene Needham Flagg, his wife's only brother, who won the D.C.M. in France and was killed at Neuve Chapelle on June 9, 1916. Mr. Gould has already endowed a bed at the Welsh Hospital at Netley in memory of his brother-in-law. Another Cardiff shipowner, Mr. Frederick Jones, with Mr. Gould, has made himself responsible for obtaining sufficient funds to endow a bed in memory of the late Mr. Edward Jordan, general manager of the Cardiff Junction Dry Docks. In appreciation of the kindness and skill he received while under treatment, Major J. A. Gibbs, D.S.O., of Penarth, who recently was killed in the war, presented the hospital with a cheque for 1,000 guineas for the endowment of a bed to be named after his boy Jack. Lastly, the wounded and sick officers now being treated in the Shand Ward have intimated their intention of endowing a bed, as a mark of their gratitude for the kindness and skill shown by the doctors and nurses.

### MR. REA'S MANIFESTO.

The task of raising the big sums necessary to complete the present development of King Edward VII.'s Hospital, Cardiff, has exercised of late many pens and voices. We have already quoted some of the humorous touches which Mr. Leonard D. Rea has thrown into his letters. Like a good hospital man, however, he is inexhaustible in resource, and among his latest letters is a really remarkable manifesto on the manysidedness of hospital work. He cunningly begins with a postscript, a beginning that he declares to be " in keeping with my origin." It announces another gift of £500. The donor is Mrs. W. H. Seager. The next passage we must quote: "My letters" (he says) "have been referred to so frequently as 'pathetic,' and as appealing so directly to sentiment, that I am sensible of failure in presenting the manysidedness of hospital work. Indeed, no one person is wise enough, or clever enough, or good enough, to explain all that a hospital means. There are thousands of people, quite uninterested in hospitals—indeed, who have never been inside one—who owe their lives, under Providence, to the skill of a doctor or the devotion of a nurse, and yet in every instance the doctor or the nurse owes to some hospital this knowledge and experience." Mr. Rea then quotes at length a passage from one of Sir James Paget's addresses to medical students in 1846, and shows that the responsibility of the doctor and the nurse falls also in its degree on every healthy person. Good as this is in itself, we quote it because it does remind us that there is no excuse for hospital appeals to be the dull documents which they often are. It is the dull dog who does not see the wealth of material at his disposal. That is one reason why a secretary, to do his work well, must be not only a man of energy, but also a man of culture. For culture is the mirror by which we can multiply our view of the world.

### THREE-HA'PENCE A WEEK.

The colliery enginemen, firemen, and mechanics, together with the North Staffordshire Miners' Federation and the National Amalgamated Union of Enginemen and Firemen, have agreed to contribute to the North Staffordshire Infirmary "as from the first make-up day in November" at the rate of 1½d. per week, to be deducted weekly from each man's money; while boys under sixteen will contribute 1d. This, together with the support given by the employers' associations, is an important and happy example of co-operation. We understand also that the smelters in one shop have agreed to give 4d. per head per week for the next twelve months to the infirmary.

### RED CROSS POSTER STAMPS.

The collector of charity stamps has few opportunities of interest in this country, and therefore we should perhaps record the statement of Mr. A. D. Richardson, who announces that he has "a few sets (six in a set) of the first authorised issue of British Red Cross Poster Stamps," the originals of which, by well-known artists, are to be preserved, we understand, in the National War Museum. The stamps, which were issued during the first month of the war, are dated 1914. The price of reproductions is 6d. for one, 1s. for three, and 2s. 6d. for seven sets. The money, which should be sent in "postal notes only," will go to the "Our Day" collection, and should be addressed to Mr. Richardson at 199 Piccadilly.

### THE STOCKPORT EXTENSION SCHEME NEAR COMPLETION.

In consequence of a varied activity (comprising the sale of flags, musical festivals, a concert, a gala, golf-club events, and so forth, a net sum of £1,116 has been obtained for Stockport Infirmary. In 1911 it was decided to commemorate King Edward VII. in Stockport by raising money for enlarging the infirmary. This scheme provided for the erection of a nurses' home, a new out-patient department, an accident-room, and a dispensary. The idea was that the existing block, comprising similar departments and a kitchen, should be transformed into a modern institution kitchen unit with stores and dining-rooms attached. The nurses' home was completed in 1914, and the new outpatient department and accident-room, after delay due to the war, will shortly be ready for use. The urgent need for these up-to-date buildings has involved increased cost, and in May of this year a special appeal was made in Stockport and district, under the patronage of his Worship the Mayor (Mr. Thomas Rowbotham), for £7,000 to meet the bank overdraft on the Building Fund, and the expenses still to be incurred. A feature of this appeal is an endeavour to provide an additional capital sum by way of endowment. So good has been the response that £9,000 has been received or promised to date. There is, then, a financial policy to which the minor expedients of flag-days and galas are subordinate. To keep control over both, Mr. Hedley Lucas, the secretary, has worked hard, and received the reward of his energy.

### A HOSPITAL AND THE WAR LOSSES COMMISSION.

The East Sussex Hospital has filed a claim with the War Losses Commission in respect of a house which has been occupied by soldiers. The hospital claims a sum of £57 9s. 8d. on the ground that it is losing a large sum annually because of the space in the house which is devoted to wounded soldiers. It is urged, briefly, that in 1915 the house was let at £100, and that the War Office only offered £19 for wear and tear. It would seem that this sum neither corresponds to the rental value of the house nor to the amount of depreciation occasioned by its occupation by the military. At the time of writing the decision is not known.

### MR. LLOYD GEORGE'S THANKS TO THE STAFFS.

The Prime Minister's public expression of thanks to "those who have undertaken hospital work in the country," coming as it does with the long list of nurses and others who have been mentioned to the Secretary of State for War in recognition of their service, is timely. It is a new identification of hospital work with the life of the nation, a tribute indeed to the hospital spirit of personal service which has made the nation one. In addressing his letter to Colonel Hepburn, commanding the 3rd Western General Hospital, the Prime Minister was careful to extend his recognition "to the medical, nursing, and other staffs of all our military hospitals," and what voluntary hospital is there today which can be excluded from this category?

### THE TERM OF OFFICE OF ABSENT HONORARIES.

The governors of the Brighton and Hove Hospital for Women and Children have decided to amend Statute 72, under which the term of service of the honorary staff has been determined. In justice to Dr. Jacomb Hood, who but for the war would have completed twenty years' service at the hospital, the statute has been amended to provide that during the continuance of the war the time spent by the honorary medical staff on military service away from Brighton shall not count as part of its member's term of office. Otherwise, for instance, Dr. Hood's term would have expired, although the last three years of it have been spent away from the institution. Since it is also agreed that in no case shall a member of the staff continue in office after the age of sixty, the spirit of the old rule is still preserved. At the same time injustice to individuals is avoided.

### CHAPLAINS AND AMBULANCE-MEN.

We can sympathise with the Bishop of London and those who approached him in the abandonment of their plan for a "parsons' ambulance corps." In an interesting public letter the Adjutant-General explained why it was impracticable. He said that the numerous "caste units" raised during the early years of the war were found very difficult to maintain, owing to the absence of reserves of the same class, and to the disinclination of all parties to accept as recruits men of a different class, or to disperse themselves among other units. The Adjutant-General further stated that the *personnel* of the Army must be kept as fluid as possible if the forces in the field were to be maintained. These are the arguments. But the proof is supplied by the Chaplain-General's announcement, made on the same day, that the waiting-list of chaplains is at this moment depleted. This settles it.

### THE JACK CORNWELL MEMORIAL COT.

The Mayor of Grimsby on October 10 unveiled the tablets over the two cots which have been endowed at Grimsby Hospital—the Jack Cornwell Memorial Cot and the Lloyd George Cot, the latter presented by Mr. Alec Black, J.P., a wealthy trawler-owner, in commemoration of what he says is the bravest civilian act of the war—the assumption by Mr. Lloyd George of the Premiership at a very critical time. Both cots are in the ward where the Jutland Battle boy hero died.

### THE SPARE BEDROOM.

What seems to be a sensible suggestion is made at an opportune moment by Dr. Barwise, the medical officer of health for Derbyshire. In view of the fact that the Government proposes to carry out great housing schemes when the war comes to an end, he suggests that advantage should be taken of this to design a type of house in which there is a bedroom with an open-air sleeping balcony, so that a delicate child or a consumptive member of the family can enjoy the open-air treatment at home. He emphasises the fact that the problem of tuberculosis is one of housing, and declares that the experience already gained of isolating advanced cases points to the necessity for there being a means of housing advanced cases at their own homes in such a way that they are no danger to the other inmates, and shelters are not always suitable in the winter for the housing of patients.

### NURSE-MIDWIVES FOR DERBYSHIRE.

Several decisions of more than passing interest and importance were taken at a recent meeting of the Derbyshire County Council. In the first place the Council undertook, in order to assist the formation of new district nursing associations for providing nurse-midwives in sparsely populated districts, to pay the annual deficit of any such association to the extent of £30 for the first year of its existence, £25 for the second, and £20 for the third year. Secondly, the Health Committee was given authority to engage during the current year six trained midwives to practise in such districts at a salary of £1 a week, the midwives to retain the fees they earned. It was stated that the original proposal to pay subsidies only was not found sufficient to induce suitable women to take up the work. Half the cost of the scheme will be borne by the Local Government Board. Thirdly, it was decided to apply to the Board for authority to provide nurses for the treatment of measles. Another important resolution was to adopt a scheme for the treatment of venereal diseases at the Derby Royal Infirmary, at a cost to the Council of £150 for the first year.

### THE LACK OF MIDWIVES IN CORNWALL.

It is in the sparsely populated counties, such as Cornwall, where the lack of midwives is most apparent. In the Duchy—it was reported at a meeting of the County Sanitary Committee held at Bodmin on October 18—there are forty-six parishes unprovided for. Dr. Clarke, the County Medical Officer, said Cornwall could do with twenty more midwives, but there was no prospect of getting them unless they trained them themselves, and then bound them to service in the county. A lady member of the committee agreed that this policy must be adopted if the maternity and child welfare scheme was to be properly carried out. The Midwives' Committee recommended the County Council to make a grant of £250 to the Cornwall Nursing Association.

### IMAGINATIVE SOLDIER-PATIENTS.

"Excuse slip, a shell has just whizzed by," writes a soldier-patient in a base hospital many, many miles away from the reach of German guns. "Talk about long-range guns!" says a R.A.M.C. writer, narrating the incident, in an amusing article contributed to the *Caledonian Medical Journal.* Another patient who had been moved to another base hospital a few miles along the coast writes: "Dear Mother, as I write this the guns are crashing [which was no doubt perfectly true!]. I have been moved five miles nearer the firing-line, but do not be alarmed." We suppose when perfect anonymity is preserved there is no harm in taking mental note of a few gems from censored correspondence. The temptation to quote the following from a patient's letter was too strong: "It's very nice to be wakened up by the nurse first thing in the morning and just put your hand out and you receive a nice cup of tea—real Old Woman's tea, plenty of everything in it, and our night nurse is the Drawn Image of our Stephen's Young Woman." We cannot refrain from retailing what a newly arrived American doctor said to the man told off to act as his batman (officer's servant): "Wall, Billy, I guess you and me's goin' to be chums!"

### LIGHT-WEIGHT SPLINTS.

At the new hut of the Surgical Requisites Association just opened in Mulberry Walk, Chelsea, the manufacture of splints and other orthopædic apparatus from *papier mâché* which the Association has carried on for over a year will be continued on a large scale for special cases. The advantages of *papier mâché* for this purpose are obvious. It is cheap to produce—old sugar-bags are one of the chief sources of supply—easy to mould upon the limb when wet, and extremely light for the wearer. Its superiority to the older appliances of wood and leather has led to a very large demand, and already workers in places as far distant as Rome, Aberdeen, and Paris are busily engaged in their manufacture. The Association has 602 branches and sub-branches throughout the country, and over seven million articles have been forwarded to the headquarters of the Guild at St. James's Palace. An exhibition of surgical appliances made under their auspices is announced at the Grafton Galleries for November 20, at the close of which an album containing the autographs of every prominent man connected with the war will be sold by public auction.

### SCHOOL DENTAL CLINICS.

Canada is alive to the importance of the proper care of children's teeth, and a system of free school dental clinics is already in operation in many cities, notably those in Eastern Canada. Winnipeg is the latest city to come into the line of progress in this respect, for it has just decided to establish four such clinics in connection with the schools. It is proposed that the clinics shall commence work during the coming school year.

### NO £100 A YEAR MASSAGE ASSISTANTS.

There is either a dearth of massage assistants or the offer of a salary of £100 a year is too little to attract them. The Plymouth Education Committee recently advertised for an assistant for remedial gymnastics and massage at a salary of £100 a year, rising by £10 a year to £140, but there was only one applicant, who, however, subsequently withdrew. Now the Education Committee is to advertise again, not mentioning any salary but merely intimating that previous experience will be taken into consideration when the committee fixes the initial salary.

### THE WATERING OF MILK.

Those who are interested in infant welfare will have noticed with thankfulness the righteous indignation shown by the Dublin Recorder when he had before him, one day last week, the case of a milk vendor who was charged with having sold milk which, in one instance, was adulterated by the addition of water to the extent of 40 per cent. Characterising this as "absolutely nothing but murder," the Recorder sentenced the defendant to a month's hard labour. On the other hand, one of the magistrates at Swansea recently announced that it was his intention to dismiss every milk prosecution that came before him until farmers were compelled to seal the cans in which the milk is conveyed to the retailers. The learned magistrate's action is probably due to the idea that milk may be abstracted and water added during the transit of the milk. Rather the onus of proving that the milk was as he had received it should be laid on the retailer.

### THIS WEEK'S DRUG MARKET.

A very fair business has been done, and prices are well maintained and in some cases considerably higher. One of the few exceptions is Cape aloes, which has changed hands at lower prices. Honey is much dearer, and a large business has been done; in view of the difficulty of obtaining sugar it might not be inadvisable for hospital buyers to take in a supply of honey even at the present extremely high rates, for any stock that may not be required for medicinal purposes can be put to other uses; it seems almost certain that prices will advance, in view of the enormously increased demand. Business has been transacted in coca-leaves at higher prices. The value of ergot of rye has an upward tendency, and the same may be said of gum benzoin. Myrrh is dearer. The value of ipecacuanha is well maintained. Rhubarb is dearer. There is a good demand for calumba root at high prices. Balsam Peru and balsam Tolu are dearer. In synthetic drugs the most interesting product at the moment is phenacetin; owing mainly to competition between manufacturers in the United States the price in America has fallen considerably, and the fall is reflected on the London market. There has been a good demand for acetanilide, and the value has an upward tendency. The supply of salicylic acid and sodium salicylate is more plentiful and prices are barely maintained. A small steady business continues to be done in quinine, and the price has a slight upward tendency.

### TO OUR READERS.

Contributions are specially invited from any of the readers of The Hospital. They should deal with topical subjects and news or incidents illustrating any department of hospital work, its progress or difficulties. They must be authenticated for the information of the Editor only. The minimum payment if published is 5s. There is no hard-and-fast rule as to space, but paragraphs of about twenty lines in length are preferred.

